# Defining and mapping the person with osteoarthritis for population studies and public health

**DOI:** 10.1093/rheumatology/ket346

**Published:** 2013-10-29

**Authors:** Elaine Thomas, George Peat, Peter Croft

**Affiliations:** ^1^Arthritis Research UK Primary Care Centre, Keele University, Keele, North Staffordshire, UK.

**Keywords:** osteoarthritis, epidemiology, prevalence, service planning

## Abstract

**Objective.** To determine population-based estimates for the prevalence of the person with OA, predicted to be the single greatest cause of disability in the general population by 2030, in order to inform the planning and commissioning of health, social care and prevention services.

**Methods.** A postal survey to all adults ≥50 years of age registered with eight general practices in the UK. Self-reported data on chronic joint pain in four body regions (hand, hip, knee, foot) and the disabling nature of the pain was collected to determine gender and age-group specific prevalence estimates of clinical OA in the joint region and in the person. Multiple imputation and weighted logistic regression was used to allow for missing data.

**Results.** A total of 26 705 mailed surveys resulted in 18 474 responses (adjusted response = 71.8%). Approximately half of the mailed population had OA in at least one of the four regions (53.23%, 95% CI 52.3, 54.1) and less than half of these had disabling OA (21.87%, 95% CI 21.2, 22.5). The more joint regions involved, the more likely that the OA was disabling. OA prevalence was higher in females and increased with age. Applied to the population of England, this yielded an estimated 3.5 million persons with disabling OA, including 1.45 million people between 50 and 65 years of age and 370 000 ≥85 years of age.

**Conclusions.** A simple approach to defining the person with OA can contribute to population comparisons, public health projections and health care needs assessments.

## Introduction

OA is a major cause of disability around the world [1]. In the UK it is the most common chronic condition seen in primary care [2], and it is predicted to be the single greatest cause of disability in the general population by 2030 [3]. The potential to prevent, control and treat OA underlines its public health importance [4].

Although OA is understood as a structural joint disease, typified radiographically by cartilage loss and bone changes, it is also a clinical syndrome of pain, stiffness and restricted mobility, and people with similar X-ray appearances can have different symptoms and degrees of disability in their daily lives. Influences on structural OA include systemic joint disease and damage (genetic, metabolic, inflammatory), local risks (undue mechanical stress, injury, childhood structural anomalies, repetitive use), age-related tissue changes (cell senescence, increased bone turnover) and function (sarcopenia, reduced proprioception) [5]. Other structural changes, such as synovitis [6], may contribute to OA pain. However, the clinical syndrome of OA is often influenced by a broader set of factors than radiographically defined OA alone. Factors such as muscle strength, mood, cognition and co-morbid illness affect joint pain and disability [7]. The burden of OA also depends on the individual context—e.g. occupation and the availability of social support or public transport [8]. This complex mixture of ageing, disease, symptoms, mobility restriction and the psychosocial environment constitutes the phenomenon of OA in populations [9, 10].

However, information about the occurrence and burden of symptomatic and disabling OA in populations is currently based on studies of disease in individual joints or of recalled diagnosis of arthritis from a health professional [9]. There is a strong case that the planning and commissioning of health, social care and prevention services for OA needs to focus on people with OA rather than on individual joint diseases [11, 12]. This article uses self-reported data from a large population-based cohort of older adults to determine population estimates of the prevalence of the person with OA in order to address this gap.

## Methods

The design was a two-stage cross-sectional postal survey of an older adult population using questionnaires [the baseline phase of the North Staffordshire Osteoarthritis Project (NorStOP)]. The two stages involved an initial Health Survey (HS) and a subsequent Regional Pains Survey (RPS). Ethical approval was from the North Staffordshire Research Ethics Committee (references 1351 and 1430). A completed returned questionnaire provided consent for inclusion.

### Study population

Full details of the study design, methods and response have been presented elsewhere [13, 14]. The sampling frame was all adults ≥50 years of age registered with eight primary care general practices in North Staffordshire, UK. The general practitioners screened out people with severe psychiatric or terminal illness from the mailing. In the UK ∼98% of the population are registered with a GP and practice registers provide a convenient frame for sampling a local population, regardless of the extent or nature of any contacts with the practice. Baseline questionnaires were mailed, and reminders were sent to non-responders after 2 and 4 weeks.

### Questionnaires

#### Stage 1—the HS questionnaire

This included information on socio-demographics [15, 16], general and mental health [17, 18], the presence of joint pain and interference of pain [17]. 

For each of the four joint regions (hand, hip, knee and foot) participants were asked, ‘Have you had any pain in your (joint region) over the last year?’ Those responding positively to any of these four questions and giving permission for further contact were mailed the RPS questionnaire.

The impact of pain was measured using a single item from the Medical Outcomes Study Short Form 12 (MOS SF-12) [‘During the past 4 weeks, how much did pain interfere with your normal work (including both work outside the home and housework?)’] [17], which has five response options, dichotomized for this analysis: (i) Pain interference—Moderately, Quite a bit or Extremely and (ii) No pain interference—Not at all or A little bit. This approach has been used in previous population-based surveys of pain [14, 19–23].

#### Stage 2—the RPS questionnaire

For each joint region, data were gathered on the duration of pain in the last 12 months (<7 days, 1–4 weeks, more than 1 month but <3 months, 3 months or more) and a joint-specific measure of pain and function [Australian/Canadian (AUSCAN) OA Hand Index for hands [24], WOMAC for hips and knees [25] and Manchester Foot Pain and Disability Index (FPDI) for feet] [26].

### Definition of OA

Three questionnaire components were used to define and characterize OA at the joint region level and the person level, i.e. presence and duration of pain in the four joint regions and pain interference using the single SF-12 item [17]. 

For each joint region (hand, hip, knee, foot), two region-level definitions were applied: (i) OA—presence of pain lasting >3 months in the last 12 months, and (ii) disabling OA—OA plus the presence of pain interference. 

The definition of OA in the person drew on these two region-level definitions as follows: (i) OA in the person—presence of OA in one or more of the four joint regions and (ii) disabling OA in the person—OA in the person plus the presence of pain interference.

At the person level, the sum of the number of painful joint regions with OA or disabling OA (from zero to four) provided a grading of the extent of OA or disabling OA in the person. We classified and estimated the extent of OA and disabling OA as (i) one or more joint regions, (ii) two or more regions, (iii) three or more regions and (iv) all four regions.

### Statistical analysis

The sample eligible for the prevalence analysis were all those not excluded at any stage of the study from either the GP screen or during the mailing. The definitions above were applied to calculate prevalence estimates of OA and disabling OA for each joint region and for the whole person, overall, by gender, by age group (50–54, 55–59, 60–64, 65–69, 70–74, 75–79, 80–84, ≥85 years) and by age group within gender.

Missing data were defined on two levels: (i) item level—questionnaire(s) had been completed but single items were missing and (ii) study level—no questionnaires were completed by the individual.

Multiple imputation using chain equations [27] was used to impute missing data at the item level using the ICE command in Stata 11 (StataCorp, College Station, TX, USA). Putative auxiliary variables for inclusion in the imputation included all the socio-demographic and general health data, and the joint region-specific measures of pain and function. A single imputation process was applied to all baseline responders to impute all baseline variables of interest using appropriate distributions (linear, logistic, ordinal and multinomial logistic regression for numerical, binary, ordinal categorical and nominal categorical variables, respectively). The number of imputations was set at 20 and imputed data sets were combined using Rubin’s combination rules [28]. The logit command was used to determine the coefficients (β) (and 95% CIs) from a logistic model from which the prevalence estimates (and 95% CIs) were calculated [prevalence = e^β^/(1 + e^β^)] for the total baseline responder population.

Weighted estimates were used to adjust for missing data at the study level, to account for any initial selective non-response*.* Information on age, gender and general practice location was available for all individuals. This information was used to determine a weight to reflect the likelihood that a person with a particular combination of age, gender and practice location would return the baseline HS questionnaire. Weighted logistic regression within the imputed data sets was performed to determine prevalence estimates (and 95% CIs) in the total baseline eligible mailed population.

### Application of prevalence estimates to standard populations

The derived prevalence estimates of the person with disabling OA were applied to the age- and gender-stratified population distribution for England, taken from the 2001 census [29], to determine the number of people >50 years of age with disabling OA in England and the number of people >50 years of age with disabling OA in an average population of 100 000 served by a general practice health care commissioning group.

## Results

After 605 exclusions before mailing, there were 26 100 adults ≥50 years of age in the eligible mailed population [54.3% female; mean age 66.0 (s.d. 10.7) years]. A total of 18 474 individuals returned their HS questionnaire. The imputation process was performed on missing items of data in these 18 474 individuals [55.8% female; mean age 66.2 (s.d. 10.2) years]. The weighted logistic regression analysis then extrapolated the results of the imputed analysis of the 18 474 HS questionnaire responders to the full initial eligible target population of 26 100 persons. Using these combined techniques, prevalence estimates were almost identical in the baseline responder population and the total baseline eligible mailed population, hence the latter data are presented.

### Prevalence estimates

Prevalence estimates at the level of the joint region in the total baseline eligible mailed population, overall and by age group, are presented in Table 1. Prevalence estimates were highest for knees and hands, twice as high for each chronic joint pain as for chronic interfering pain and higher in females than males for all four joint regions. Estimates generally increased with age, particularly for chronic, interfering pain.

**Table 1:** Prevalence estimates for OA in the joint region: overall and by gender

	Joint region							
	Hand		Hip		Knee		Foot	
	OA	Disabling OA	OA	Disabling OA	OA	Disabling OA	OA	Disabling OA
Overall	26.54	12.56	19.16	10.77	30.65	14.91	23.15	11.65
	(25.8, 27.3)	(12.0, 13.1)	(18.5,19.8)	(10.3, 11.3)	(29.9, 31.4)	(14.3, 15.5)	(22.4, 23.9)	(11.1, 12.2)
Gender								
Female	30.53	14.66	21.84	12.49	33.06	16.47	26.67	13.52
	(29.5, 31.5)	(13.9, 15.4)	(21.0, 22.7)	(11.8, 13.2)	(32.0, 34.1)	(15.7, 17.3)	(25.7, 27.7)	(12.8, 14.3)
Male	21.79	10.06	15.96	8.72	27.77	13.03	18.96	9.42
	(20.8, 22.8)	(9.4, 10.8)	(15.0, 17.0)	(8.1, 9.4)	(26.7, 28.9)	(12.3, 13.9)	(18.0, 20.0)	(8.7, 10.2)

Values within brackets are 95% confidence intervals.

Prevalence estimates for OA in the person are shown in Table 2. Approximately half of this population had OA in at least one of the four joint regions [prevalence 53.23% (95% CI 52.3, 54.1)], which was more than twice the prevalence of disabling OA in the person [21.87% (21.2, 22.5)]. The proportional difference between OA and disabling OA estimates decreased as the extent of OA increased, indicating that the more joint regions that were involved, the more likely that person reported pain interference. Estimates of OA and disabling OA for persons reporting pain in all four joint regions were therefore quite close [OA: 4.15% (3.8, 4.5) and disabling OA: 3.27% (3.0, 3.6)].

**Table 2:** Prevalence estimates for OA in the person: overall and by gender and age group

	Definition of OA							
	1–4 regions		2–4 regions		3–4 regions		All 4 regions	
	OA	Disabling OA	OA	Disabling OA	OA	Disabling OA	OA	Disabling OA
Overall	53.23	21.87	28.75	15.73	13.37	9.02	4.15	3.27
	(52.3, 54.1)	(21.2, 22.5)	(28.0, 29.5)	(15.2, 16.3)	(12.8, 14.0)	(8.6, 9.5)	(3.8, 4.5)	(3.0, 3.6)
Female	57.39	23.85	32.95	18.07	16.35	10.99	5.41	4.23
	(56.3, 58.5)	(23.0, 24.7)	(31.9, 34.0)	(17.3, 18.9)	(15.5, 17.3)	(10.3, 11.7)	(4.9, 5.9)	(3.8, 4.7)
Male	48.28	19.51	23.74	12.98	9.82	6.67	2.65	2.13
	(47.0, 49.6)	(18.6, 20.4)	(22.7, 24.8)	(12.2, 13.7)	(9.1, 10.6)	(6.1, 7.3)	(2.3, 3.1)	(1.8, 2.5)
Age group, years								
50–54	46.03	15.26	21.21	10.23	9.04	5.76	2.94	2.31
	(43.9, 48.2)	(13.9, 16.8)	(19.5, 23.0)	(9.0, 11.6)	(7.8, 10.4)	(4.9, 6.8)	(2.3,3.8)	(1.7, 3.0)
55–59	42.35	16.76	25.53	12.29	12.27	7.93	4.01	3.31
	(47.2, 51.3)	(15.3, 18.3)	(23.8, 27.4)	(11.0, 13.7)	(10.9, 13.7)	(6.9, 9.1)	(3.3, 4.9)	(2.7, 4.1)
60–64	52.50	19.95	28.74	14.88	12.97	8.27	4.17	3.07
	(50.6, 54.3)	(18.6, 21.4)	(27.2, 30.4)	(13.7, 16.2)	(11.8, 14.2)	(7.4, 9.3)	(3.5, 5.0)	(2.5, 3.8)
65–69	54.77	20.77	30.82	15.23	14.16	8.98	4.26	3.27
	(52.6, 56.9)	(19.1, 22.5)	(28.8, 32.9)	(13.8, 16.8)	(12.7, 15.8)	(7.7, 10.4)	(3.4, 5.3)	(2.5, 4.2)
70–74	55.42	23.01	30.17	16.54	14.67	9.77	4.26	3.13
	(53.4, 57.5)	(21.5, 24.6)	(28.4, 31.1)	(15.2, 18.0)	(13.3, 16.2)	(8.6, 11.0)	(3.5, 5.1)	(2.5, 3.9)
75–79	56.60	26.04	31.81	19.21	15.05	11.01	4.70	3.93
	(53.8, 59.4)	(23.9, 28.2)	(29.3, 34.4)	(17.3, 21.3)	(13.2, 17.1)	(9.4, 12.8)	(3.7, 6.0)	(3.0, 5.1)
80–84	58.93	31.79	34.11	22.28	16.09	11.98	4.74	4.06
	(56.2, 61.7)	(29.5, 34.2)	(31.5, 36.8)	(20.2, 24.5)	(14.0, 18.4)	(10.3, 13.9)	(3.7, 6.1)	(3.1, 5.3)
≥85	62.29	38.83	35.56	26.59	16.76	13.61	4.74	4.53
	(58.0, 66.4)	(35.2, 42.5)	(31.8, 39.5)	(23.3, 30.2)	(14.0, 20.0)	(11.2, 16.5)	(3.7, 6.1)	(3.2, 6.4)

Values within brackets are 95% confidence intervals.

**Fig. 1:**
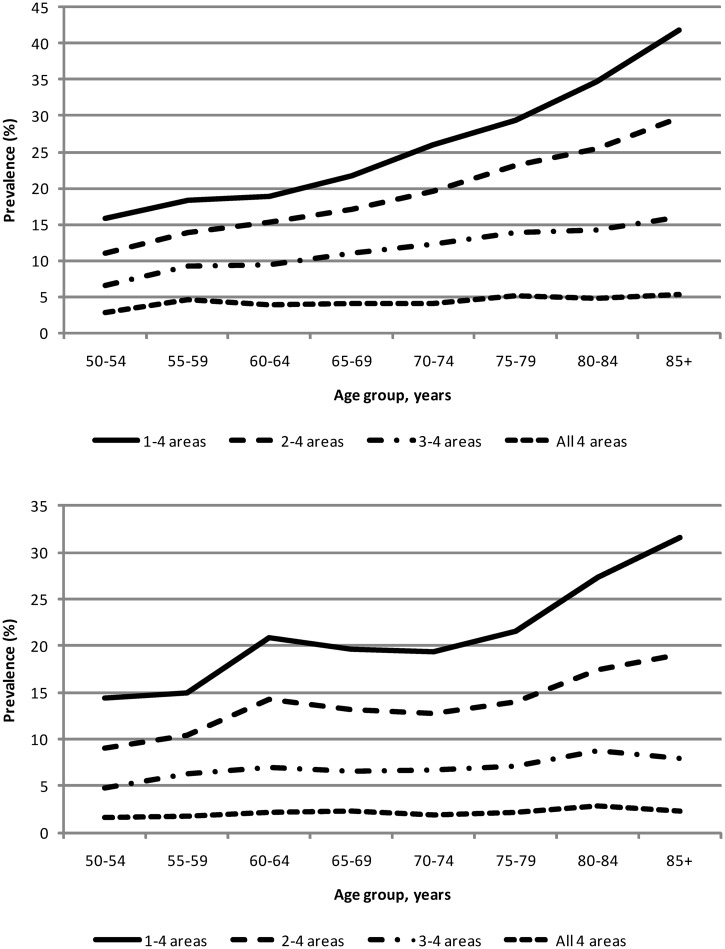
Prevalence estimates for definitions of disabling clinical OA in the person. (**A**) Females by age group. (**B**) Males by age group.

The prevalence of OA in the person was higher in females than males and generally increased with age (Tables 2 and 3, Fig. 1A and B). Almost 25% of females >50 years of age have disabling OA, increasing to 39% in females >85 years of age. The proportion of persons with OA increases with age regardless of the presence of disability or the number of sites affected. At any age >50 years, the prevalence of OA is greater for persons reporting multiple region involvement than for those with single-region pain only.

**Table 3:** Population estimates for disabling OA in the person: England and an average-sized commissioning group

	Population data for prevalence of disabling OA	
	**England** (*n* = 49 138 831)	**Average-sized commissioning group** (*n* = 100 000)
Overall	3 510 378	7144
Gender		
Female	2 096 555	4267
Male	1 413 823	2877
Age group, years		
50–54	514 610	1047
55–59	465 233	946
60–64	477 204	971
65–69	447 165	910
70–74	448 241	912
75–79	431 142	877
80–84	353 914	720
≥85	372 869	759

### Estimated numbers, using the English population and commissioning groups as an example

Applying the prevalence estimates to population data suggests 3 510 378 persons ≥50 years of age have disabling OA in England. In a typical unit of population of 100 000 people for health services commissioning in England, slightly more than 7000 persons ≥50 years of age are estimated to have disabling OA (Table 3).

## Discussion

This article concerns the clinical syndrome of OA and presents a practical approach to defining the person with OA and the person with disabling OA for population needs assessment. These definitions are not intended to replace clinical diagnosis of individual patients in everyday practice, but we argue that they do provide a useful approach to classifying persons for epidemiological and planning purposes. Their application in a self-administered population survey shows that most people with OA have pain in more than one joint region and proportionately more women than men have OA. Although joint pain generally increases with age, the sharpest increase occurs among people who have pain that interferes—39% of persons ≥85 years of age compared with 15% of persons age 50–54 years. However, these figures also mean that OA is not an inevitable consequence of ageing and prevention is a plausible target for health care.

We explored the validity of our survey estimates. There were missing data in the returned questionnaires. We investigated this by using imputation based on a range of survey information. There was also a 29% non-response to the survey. We adjusted for this by applying weighted estimates from the responder population to the age, gender and GP practice structure of the target surveyed population. Changes in estimates following these combined approaches were minimal.

### The definition of clinical OA in populations

An important concern to address is our use of self-reported joint pain and interference with daily life in an older population as the basis for defining OA. 

Estimates of the global burden of disease and local population-based health care needs assessments related to OA have traditionally focused on radiographic definitions of OA at specific joint sites [30, 31]. Although this is reasonable for aetiological studies (e.g. what causes structural OA in the hip and how might it be prevented) and in helping treatment decisions in some individual patients (e.g. the need for an X-ray prior to knee replacement surgery), this is insufficient for even targeted assessments of health care needs at a population level (e.g. how many people have hip OA sufficiently severe for joint replacement), since decisions related to a structurally focused treatment such as joint replacement are informed by the degree of interference with daily life as well as by the severity of radiographic change [10]. Hence the focus in recent studies has shifted towards pain and disability at specific joint sites, with or without radiographic measures, i.e. towards clinical OA as the condition of interest [9, 10].

Further justification for using a symptom definition for population purposes is provided by recent work [32] which, by carefully adjusting for confounding, has identified a much closer association between the severity of radiographic features of OA at the knee and pain in that joint compared with previous reports.

This then leaves the final argument for the choice of joint pain to represent clinical OA in population studies resting on the assumption that OA is the most common cause of pain in this age group. There is evidence to support this assumption. In a clinical assessment substudy of NorStOP (including X-rays), the proportion of persons with chronic hand pain and pain interference who had definite radiographic hand OA was 81%, and the equivalent figure for the knee was 78%, whereas the number of people with a joint disease other than OA in their medical record was 16 out of >800 persons with knee pain [33] and 28 out of >600 persons with hand pain [34].

Our conclusion is that evidence from a range of sources suggests that the phenotype of self-reported joint pain and pain that interferes in persons ≥50 years of age sufficiently reflects other recognized OA phenotypes (radiographic change, use of the label of OA in primary care) to be acceptable as the basis for population measures of the person with OA. As with many public health measures, this definition is crude, and a degree of misclassification has to be accepted, but it fits a particular purpose of estimating population burden. It may be less appropriate for assessing or evaluating the individual patient.

### The definition of OA in the whole person

Regardless of how OA itself is defined, prevalence estimates of OA in specific joints do not provide a clear basis for informing health and social care needs and preventive services for all persons with OA since, as confirmed here, most people with OA have problems at more than one joint site and core treatment for OA (pain relief, exercise, weight reduction, self-management) is similar regardless of the joint location [4]. A recent systematic review highlighted that measuring OA in the population has largely relied on a recalled diagnosis of arthritis or OA from a health professional [9]. It found variability of prevalence estimates arose from different measures of the problem (recalled diagnosis, radiographic, symptom-based), and the accompanying editorial [7] called for more attention on the person with OA. However, for specific questions about health care needs, different definitions and analyses may be needed [31]. A specific example would be the estimation of the number of persons requiring a joint replacement or the potential costs of joint replacement surgery, where the approach proposed here would not be appropriate on its own and would need supplementation to provide estimates at a joint-specific level and include information on radiographic severity.

One potential concern is that our definition of the person with OA includes the foot. Textbook definitions focus on the hand, knee and hips as major OA sites, but accept that any synovial joint may be affected by radiographic and clinical syndromes. The frequency of OA in joints other than the foot, however, is low compared with the hand, knee and hip, whereas the proportion of foot pain in older people that could be OA is unclear. To investigate this we recalculated disabling OA prevalence in the person excluding the foot. This figure was 20.78% (95% CI 20.2, 21.4), very similar to the prevalence including the foot (21.87%; Table 2). This means the foot does not add to the definition of disabling OA in the person. However, inclusion of the foot does contribute to grading of the number of joint regions involved and we retained it in the definition of the person with OA.

Our figures for disabling OA in the person are a little lower than estimates based on the ACR criteria using combined self-report, radiographic and clinical assessment data [35, 36]. These studies either did not estimate prevalence in the person or did so by adding up prevalence figures for individual joints, which inevitably results in overestimates.

Primary care data sets with clinician diagnostic labels provide an alternative resource for estimating population prevalence of the person with OA. Our estimates for disabling OA are rather lower than the 10-year period prevalence of diagnosed OA reported from an analysis of persons with OA in a primary care clinical database [37], which may reflect the contrast between currently troublesome OA and OA intermittently troublesome over a period. However, these contrasting approaches to determining the population prevalence of disabling OA—self-report *vs* clinical consultation history—do seem to provide compatible and comparable estimates of OA in the person.

### Conclusion

In summary, we propose that the definition and approach developed here provides an appropriate basis for estimating the number and distribution of persons with OA in local, regional and national populations, and for comparison between such populations. Three dimensions, easily captured by self-complete questions, identify subgroups of increasing severity—chronicity and pain interference provide the core definitions of disabling OA, and the number of joint regions is a simple measure of ‘how much OA have you got?’, which has associations with other measures of societal and personal impacts of OA [38].

Research on interventions for persons with co-morbid chronic diseases has highlighted the need to balance condition-specific management (e.g. diabetic control, joint replacements, heart failure therapy) with interventions common to many different chronic conditions (e.g. weight reduction, physical activity, positive cognitions, anti-depression therapies) [39, 40]. A focus on the person with OA will help to integrate these two approaches.

**Rheumatology key messages**

- There is a need for more attention on people with OA.
- Twenty per cent of people reported disabling OA, which was higher in females and those of older age.
- An estimated 3.5 million persons have disabling OA in the UK.
